# Fractal Gait Patterns Are Retained after Entrainment to a Fractal Stimulus

**DOI:** 10.1371/journal.pone.0106755

**Published:** 2014-09-15

**Authors:** Christopher K. Rhea, Adam W. Kiefer, Matthew W. Wittstein, Kelsey B. Leonard, Ryan P. MacPherson, W. Geoffrey Wright, F. Jay Haran

**Affiliations:** 1 Department of Kinesiology, University of North Carolina at Greensboro, Greensboro, North Carolina, United States of America; 2 Division of Sports Medicine, Cincinnati Children's Hospital Medical Center, Cincinnati, Ohio, United States of America; 3 Department of Pediatrics, College of Medicine, University of Cincinnati, Cincinnati, Ohio, United States of America; 4 Center for Cognition, Action & Perception, Department of Psychology, University of Cincinnati, Cincinnati, Ohio, United States of America; 5 Department of Physical Therapy, Temple University, Philadelphia, Pennsylvania, United States of America; 6 Department of Bioengineering, Temple University, Philadelphia, Pennsylvania, United States of America; 7 Biomedical Research & Operations Department, Navy Experimental Diving Unit, Panama City Beach, Florida, United States of America; Scientific Institute Foundation Santa Lucia, Italy

## Abstract

Previous work has shown that fractal patterns in gait can be altered by entraining to a fractal stimulus. However, little is understood about how long those patterns are retained or which factors may influence stronger entrainment or retention. In experiment one, participants walked on a treadmill for 45 continuous minutes, which was separated into three phases. The first 15 minutes (pre-synchronization phase) consisted of walking without a fractal stimulus, the second 15 minutes consisted of walking while entraining to a fractal visual stimulus (synchronization phase), and the last 15 minutes (post-synchronization phase) consisted of walking without the stimulus to determine if the patterns adopted from the stimulus were retained. Fractal gait patterns were strengthened during the synchronization phase and were retained in the post-synchronization phase. In experiment two, similar methods were used to compare a continuous fractal stimulus to a discrete fractal stimulus to determine which stimulus type led to more persistent fractal gait patterns in the synchronization and post-synchronization (i.e., retention) phases. Both stimulus types led to equally persistent patterns in the synchronization phase, but only the discrete fractal stimulus led to retention of the patterns. The results add to the growing body of literature showing that fractal gait patterns can be manipulated in a predictable manner. Further, our results add to the literature by showing that the newly adopted gait patterns are retained for up to 15 minutes after entrainment and showed that a discrete visual stimulus is a better method to influence retention.

## Introduction

Gait consists of a series of strides that naturally and rhythmically vary from stride-to-stride. While this phenomenon has been known for over a century [1], it has often been relegated as imprecise motor control—a position supported by numerous clinical populations that demonstrate an increase in variability in stride time intervals compared to healthy adults [2], [3], [4]. However, research over the past three decades examining the properties of adaptive and functional biological systems has challenged the traditional view of stride interval variability by showing that healthy and clinical populations may present with similar variability in their rhythms, despite having different functional behaviors [5], [6], [7], [8], [9].

All biological rhythms exhibit some level of variability, and while some of these systems remain adaptive and functional, others are maladaptive and dysfunctional. The importance of an adaptive locomotor system cannot be understated as it is constantly evolving to meet imposed challenges from constraints on the person (e.g., neurological conditions), task (e.g., walking and talking), or environment (e.g., walking on ice). Accordingly, risk of injury increases if the person is not able to adapt their gait to one or more of the aforementioned constraints. Thus, the ability to exhibit adaptive gait is a desirable characteristic in order to avoid negative outcomes.

Locomotor adaptability has been demonstrated to be closely tied to the variability of stride-to-stride intervals [10], [11]. Traditionally, variability of locomotor behavior has been measured through summary metrics (e.g., standard deviation and coefficient of variation) that index the *magnitude of variability* in the behavior of the system. However, twenty years ago, researchers first began to demonstrate that a pathological system may have the same magnitude of variability as a healthy system, while the structure of variability differed [12]. This observation led to the postulate that the structure of variability in a system's behavior may reflect the system's inherent flexibility; that is, the system's ability to exhibit adaptive, functional behavior [9], [10], [11], [13], [14]. More specifically, the rhythmic variability inherent to these systems also exhibited fractal scaling (i.e., patterns of variability at one time scale are similar to those found at other time scales). Thus, more recently, metrics that index the *structure of variability* have gained favor in the literature because of their ability to quantify the dynamic, time-evolving nature of the locomotor system's rhythmic behavior.

One way that the variability of these locomotor rhythms has been quantified is through a technique called detrended fluctuation analysis (DFA). DFA was developed to quantify long-range correlations as a means to index repeating patterns at different time scales [15]. The alpha (α) value derived from DFA describes the strength of the long-range correlations and typically ranges from 0.5 (no long-range correlations or *randomness*) to 1.0 (strong long-range correlations or *persistence*). Hausdorff and colleagues used DFA to show that persistence is observed in the stride-to-stride intervals of young healthy adults and a shift toward randomness is observed when the agent is constrained by pathology or natural aging [16], [17], [18], [19]. This finding has been extended to show that a shift toward a more random gait pattern is observed when a constraint is imposed on the person, task or environment [20], [21], [22], [23], [24], and may partially account for an increased rate of falls in many populations exhibiting this behavior [10], [25].

One way to enhance current clinical practice is to incorporate gait variability training. Specifically, the development of new interventions to change gait variability patterns would be a unique way to potentially restore functional gait behavior [26]. Our previous work has shown that fractal patterns in gait can be altered when participants synchronize their stride-to-stride intervals to a visual metronome (flashing square on a screen) while they walk on a treadmill [27]. The intervals between flashes of the visual metronome were not consistent; rather, they exhibited a variety of fractal patterns. Thus, by altering the fractal patterns of the visual stimulus and requiring the participant to synchronize their heel strike with the stimulus, our results indicated that the fractal structure in stride-to-stride intervals could be shifted toward increased persistence or randomness. The findings of our work are supported by similar results when a fractal auditory stimulus is used [20], [28], [29], [30], and all of these studies present a similar theme; fractal gait patterns can be altered when synchronizing gait to a fractal stimulus. The next logical question, then, is what happens to the gait patterns when the stimulus is removed? Do the new fractal gait patterns remain or do they return to baseline levels? Hove et al. examined the carry-over effects in Parkinson's patients after three minutes of gait synchronization to a fractal auditory stimulus, but the retention trial only lasted three minutes [20]. Uchitomi et al. examined the retention of gait patterns in Parkinson's patients across four days, but also only examined three minute gait trials [30]. Longer retention tests and the identification of factors that influence retention are necessary to develop protocols that may enhance locomotor rehabilitation.

The purpose of this study was two-fold. The first experiment was designed to test whether fractal gait patterns are retained for up to 15 minutes after entraining gait to a fractal stimulus. Entrainment in this study refers to synchronizing gait patterns to a stimulus. It was hypothesized that the gait patterns after the entrainment phase would be similar to those observed during entrainment. In the second experiment, we tested the influence of a continuous (i.e., visual information for synchronization was available nearly the entire time) versus a discrete (i.e., visual information for synchronization was available only at heel strike) fractal stimulus on fractal gait patterns during the synchronization and post-synchronization (i.e., retention) phases. In this experiment, we hypothesized that the continuous stimulus would lead to more a persistent gait pattern in the synchronization phase. It was also predicted that individuals would exhibit fractal gait patterns more similar to the stimulus pattern in the post-synchronization phase when the continuous stimulus was employed. A brief outline of each experiment and the respective methods follows.

## Experiment 1 – Determining whether Fractal Gait Patterns Are Retained after Entrainment

This experiment was designed to replicate and expand our previous work using a visual stimulus exhibiting fractal timing patterns as a mechanism for individuals to develop a desired change in fractal timing patterns of gait [27]. This was accomplished by instructing the participants to entrain their gait cycle to the visual stimulus. Our previous work showed that fractal gait patterns in young, healthy adults could be moved toward more random (i.e., toward DFA α = 0.5) or persistent (i.e., toward DFA α = 1.0) patterns when synchronizing their gait cycle to a visual stimulus exhibiting random or persistent patterns, respectively. The logical progression of this work is to determine if those patterns are retained after healthy adults train with a fractal stimulus. We note that the healthy participants in this study were presumed to exhibit adaptive, functional behavior. Thus, requiring them to shift from their baseline behavior (DFA α = 0.75) toward a more persistent behavior (DFA α = 1.0) could be interpreted as shifting a healthy system into a maladaptive system. This is congruent with perspective that interprets any change in behavior (i.e., an increase or a decrease in DFA α) as a shift toward a maladaptive system [10], [31]. However, most clinical populations exhibit a shift toward a more random gait pattern (DFA α = 0.5), so Experiment 1 was designed to be a proof-of-concept study to determine whether more persistent behavior would be adopted when entraining to a fractal stimulus, regardless of the starting point of each participants' baseline behavior.

### Materials and Methods

#### Participants

Twelve young healthy adults (7 females and 5 males, age: 23.5±4.5 yrs; height: 1.67±0.09 m; mass: 64.4±8.9 kg) participated. All participants were screened for any neurological conditions or structural injuries that would affect their gait.

#### Ethics Statement

The University of North Carolina at Greensboro institutional review board approved all procedures, and all participants signed an informed consent form prior to participation.

#### Procedure

Participants walked at a self-selected walking speed (*M* = 1.08±0.03 m/s) on a treadmill for a total of 45 minutes continuously, which included three 15 minute phases. In the first 15 minutes (pre-synchronization phase), participants walked at their preferred speed, which served as a baseline. In the next 15 minutes (synchronization phase), the participants synchronized their gait cycle to a visual metronome that exhibited persistence (DFA α = 0.98). As in our previous work [27], the visual metronome consisted of a red flashing square that was projected in front of the treadmill and participants were asked to synchronize to the metronome by being at right heel contact when the red square flashed. The average interval between red square flashes was 1.00±0.07 sec. In the last 15 minutes (post-synchronization phase), the metronome was taken away and the participants were asked to walk naturally, just as they did in the pre-synchronization phase. They were not told to attempt to reproduce the gait timing patterns from the synchronization phase, as our goal was to determine what behavior naturally emerged after entrainment to the fractal stimulus.

Twelve reflective markers were attached to the participant and affixed bilaterally on the lower limbs at the mid-thigh, knee, mid-shank, ankle, heel, and toe. Gait kinematics were captured via a Qualisys 3D Motion Capture system at 200 Hz (Qualisys, Gothenburg, Sweden). Even though subjects were asked to synchronize their right heel strike to the visual metronome, we found no difference between legs in our previous work [27], so only the right leg was used in the current analysis. The knee angle in the sagittal plane was then calculated with customized Matlab routines at each time point (1/200^th^ sec) (Mathworks, Natick, MA). Next, the time interval between each peak knee flexion was calculated using a custom Matlab algorithm, creating a stride-to-stride interval time series. Each 45 minute time series was separated into three phases of 15 minute time series within a complete trial: (1) pre-synchronization, (2) synchronization, and (3) post-synchronization. The dynamics of each stride-to-stride interval time series within each phase was analyzed using DFA to index baseline gait dynamics before the metronome (pre-synchronization phase), the degree to which gait dynamics were altered when walking to the metronome (synchronization phase), and the residual effect of the altered gait dynamics when the metronome was removed (post-synchronization phase).

The details of DFA have been outlined elsewhere [15], [32] and in our previous work [27]. Briefly, the time series is first integrated and then divided in boxes (i.e., time durations) of equal size. Next, the data within each box is detrended by applying a line of best fit to the data and determining the deviation of each data point from the line. The average deviation about the line within each box is calculated throughout the time series and then repeated for a variety of box sizes (*n* = 4 to *n* = 1/4 × number of data points). A log-log plot is then created by plotting the log of the box size *n* on the x-axis and the average deviation within each box size on the y-axis. Lastly, a line of best fit is applied to the plot and the slope of the line (α) corresponds to the strength of the long-range correlation. Typical DFA α values for stride-to-stride intervals in gait hover around 0.75. DFA α near 0.5 indicates a more random pattern, whereas values near 1.0 are tending toward persistence.

#### Statistics

All statistics calculated with the IBM SPSS Statistics Package (version 18, IBM Corporation, New York). Summary statistics (mean and standard deviation) and the fractal structure (DFA α) of the stride-to-stride intervals were examined for each phase. Tests of normality (skewness, kurtosis, and Kolmogorov-Smirnov) indicated all dependent variables were normally distributed. A separate repeated measures analysis of variance (ANOVA) was used to examine each dependent variable (*p*≤.05). Follow-up Bonferroni corrected *t*-tests were used when appropriate.

### Results

#### Summary statistics

**Figure 1:**
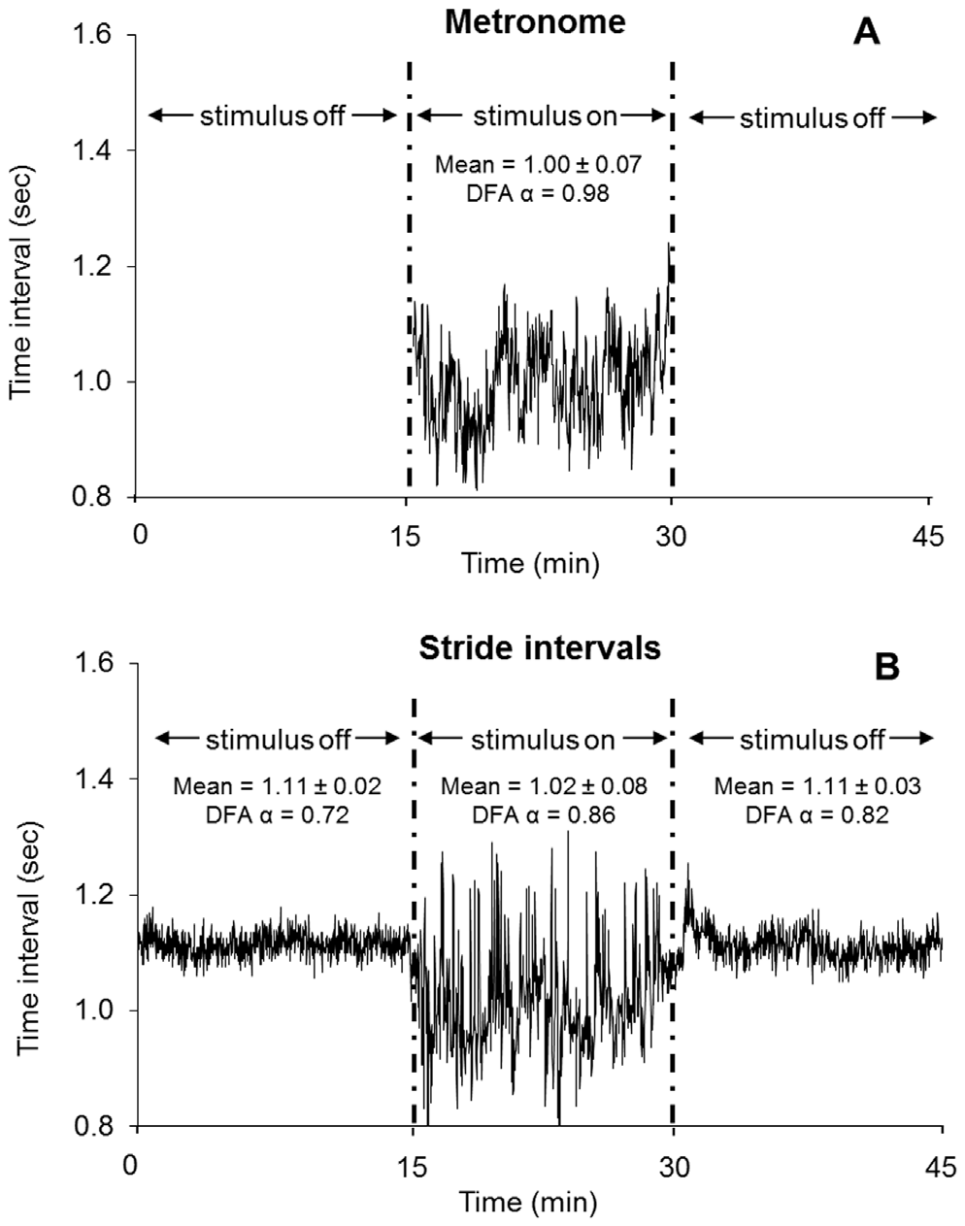
Time series of the stimulus and stride intervals in Experiment 1. The fractal time series used to drive the metronome (A) and one participant's stride interval time series before, during, and after synchronizing with the metronome (B). The mean, standard deviation, and DFA α for each phase is presented. DFA α increased the synchronization phase and remained elevated during the post-synchronization phase.

**Figure 2:**
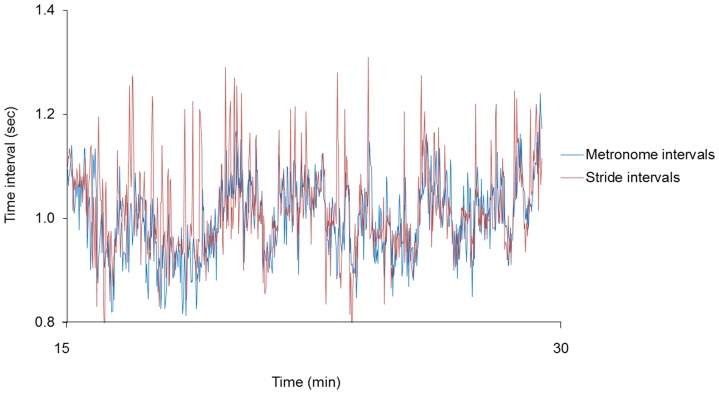
Synchronization phase time series for the metronome and stride intervals in Experiment 1. The fractal pattern of the metronome time series that prescribed the gait patterns is depicted in blue and the actual stride interval time series during the synchronization phase is depicted in red. Although the stride interval time series had greater variability magnitude, similar underlying structure is observed in both time series.

**Figure 3:**
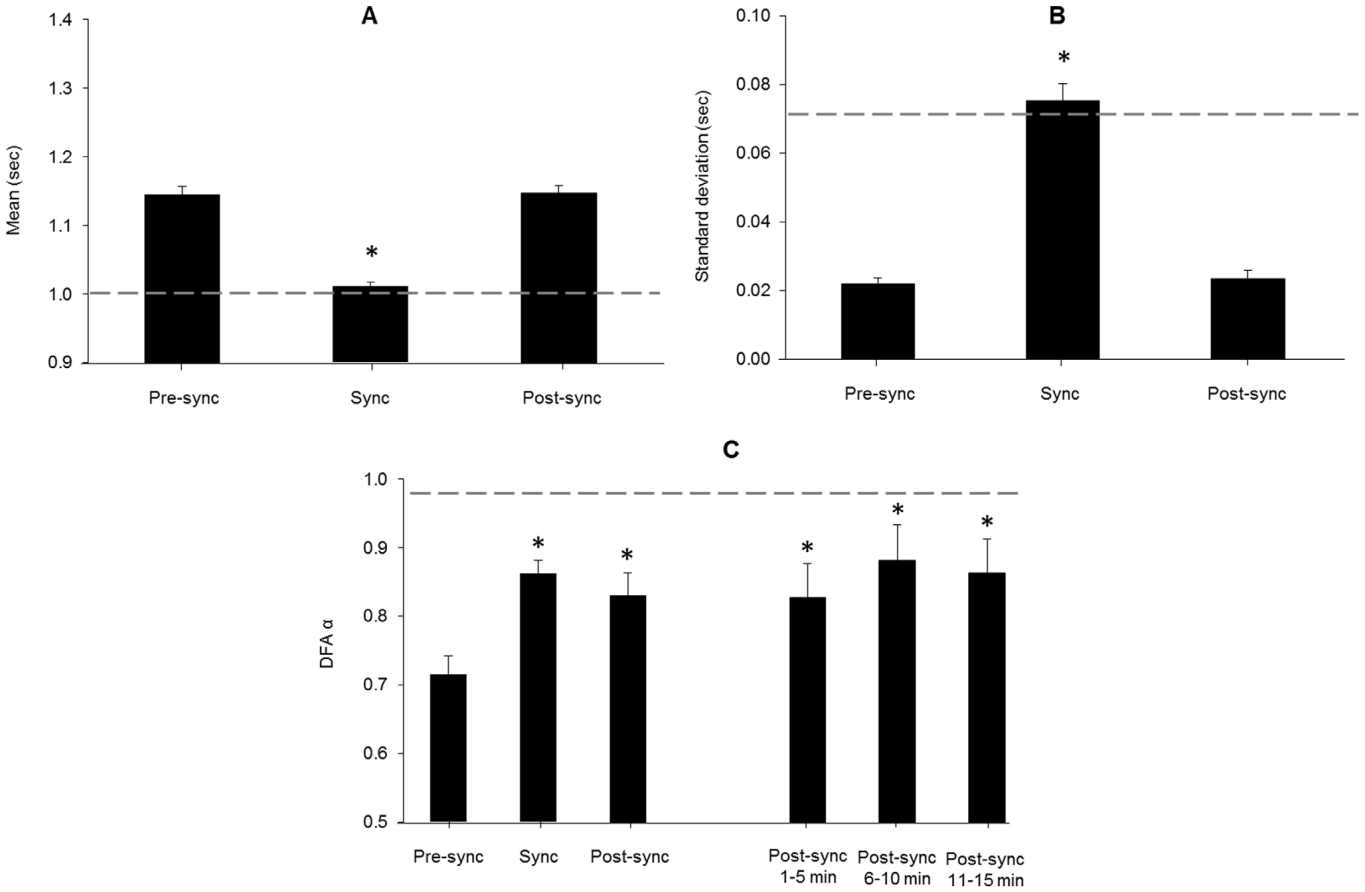
Mean, standard deviation, and DFA α of the stride interval time series in Experiment 1. A significant decrease in mean (A) and increase in standard deviation (B) was observed during the synchronization phase. The dashed gray line indicates the mean (1.00 sec) and standard deviation (0.07 sec) of the fractal stimulus that was used during the synchronization phase. Error bars represent standard error. Asterisks indicate the sync phase was significantly different relative to the pre- and post-sync phases for mean and standard deviation. A significant increase in DFA α (C) was observed in the synchronization phase, which was retained in the post-synchronization phase. Follow-up analyses showed that the post-synchronization elevated values were not only due to immediate retention. Rather, all three 5 minute epochs in the post-synchronization phase exhibited an elevated DFA α value. The dashed gray line indicates the DFA α value (0.98) of the fractal stimulus that was used during the synchronization phase. Asterisks indicate the sync and post-sync phases were significantly elevated relative to the pre-sync phase, and that the post-sync 1–5, 6–10, and 11–15 phases were not different from each other.

An example of the stride-to-stride interval time series for the 45 minute trial encompassing the three phases is in Figure 1. The middle 15 minutes is expanded in Figure 2 to provide a comparison of the prescribed fractal pattern (metronome intervals) and the corresponding gait behavior (stride intervals) during the synchronization phase. A main effect of phase was observed for the mean, *F*(2,22) = 74.8, *p*<.001, partial η^2^ = .87, and standard deviation, *F*(2,22) = 97.4, *p*<.001, partial η^2^ = .90, of the stride-to-stride intervals. Follow-up tests indicated that the mean and standard deviation in the pre-synchronization and post-synchronization phases were not different, but the synchronization phase had a significantly lower mean and higher standard deviation in the synchronization phase (*p*<.001; Figure 3).

#### Fractal structure

A main effect of phase was observed for DFA α, *F*(2,22) = 10.5, *p* = .001, partial η^2^ = .49, and follow-up tests indicated that DFA α significantly increased when comparing the pre-synchronization phase (0.72±0.09) to the synchronization phase (0.86±0.07; *p*<.001). DFA α remained high during the post-synchronization phase (0.83±0.12), and was not significantly different from the synchronization phase (*p* = .380). However, DFA α was significantly higher in the post-synchronization phase compared to the pre-synchronization phase (*p*<.001; Figure 3). To determine if the DFA α values during the post-synchronization phase were driven by the initial stride-to-stride interval dynamics in the phase, the 15 minute time series was further separated into three 5 minute, non-overlapping time series. These shortened time series are similar to the duration of the retention time series examined by Hove et al. [20] and Uchitomi et al. [30], which allowed for a more direct comparison between studies. However, those studies only examined retention for 3 minutes following the gait training, whereas our study extended the retention phase to 15 minutes, allowing for three 5 minute non-overlapping time series to be examined. We elected not to shorten the time series to less than 5 minutes, as the patterns indexed by DFA may be inaccurately identified in short time series. No difference in DFA α was observed between the 5 minute intervals, *F*(2,22) = 1.27, *p* = .301, partial η^2^ = .10, indicating that similar fractal structure in the gait dynamics was observed throughout the 15 minute post-synchronization (i.e., retention) phase (Figure 3).

## Experiment 2 – Continuous versus Discrete Fractal Stimuli: Determining Which Method Is Better for Fractal Gait Retention

Experiment 1 demonstrated that fractal gait patterns are retained after synchronizing to a fractal visual stimulus. However, the results from our previous work [27] and Experiment 1 indicate that participants are not able to fully match the fractal characteristics of the visual stimulus. In both experiments, participants were instructed to synchronize their gait to a fractal visual stimulus exhibiting a variability pattern of DFA α = 0.98. In both cases, participants were not able to fully produce the fractal pattern exhibited by the stimulus, but did increase the persistence in their gait patterns during the synchronization phase (DFA α = 0.87±0.06 in [27] and 0.86±0.07 in Experiment 1 of the current study). The same discrete stimulus (flashing red square) was used in both experiments to prescribe the desired gait patterns, and in the absence of continuous visual information, the task required a level of anticipation of when the next square will flash in order to match up the right heel strike to the visual display. Previous work has shown that synchronization performance increases when a continuous stimulus is used compared to a discrete stimulus [33]. Thus, Experiment 2 was designed to investigate if gait patterns could be more precisely shifted when using a continuous fractal stimulus compared to a discrete fractal stimulus during the synchronization phase and if those more persistent patterns were retained in the post-synchronization phase.

### Materials and Methods

#### Participants

Fifteen young healthy adults (7 females and 8 males, age: 24.7±5.2 yrs; height: 1.77±0.10 m; mass: 75.5±11.5 kg) participated, none of whom participated in Experiment 1. All participants were screened for any neurological conditions or structural injuries that would affect their gait.

#### Ethics Statement

The University of North Carolina at Greensboro institutional review board approved all procedures, and all participants signed an informed consent form prior to participation.

#### Procedure

Participants attended two data collection sessions over two separate days. Similar to Experiment 1, participants walked for an extended period of time that was separated into three phases. The total time of the two daily sessions in Experiment 2 was shortened to 30 minutes. This led to three 10 minute phases, which still allowed for approximately 500 strides within each phase. In both sessions, participants walked at a self-selected walking speed (0.93±0.09 m/s) on a treadmill for a total of 30 minutes continuously. For the first 10 minutes, participants walked at their preferred speed, and this served as a baseline trial (pre-synchronization phase). During the next 10 minutes, the participants synchronized to a visual stimulus that exhibited persistence in the inter-beat intervals (DFA α = 0.98, synchronization phase). For the last 10 minutes, the visual stimulus was removed and the participants were told to continue walking (post-synchronization phase). Just as in Experiment 1, the participants were told to walk naturally after the stimulus was removed (i.e., they were not told to attempt to reproduce the fractal patterns from the synchronization phase).

**Figure 4:**
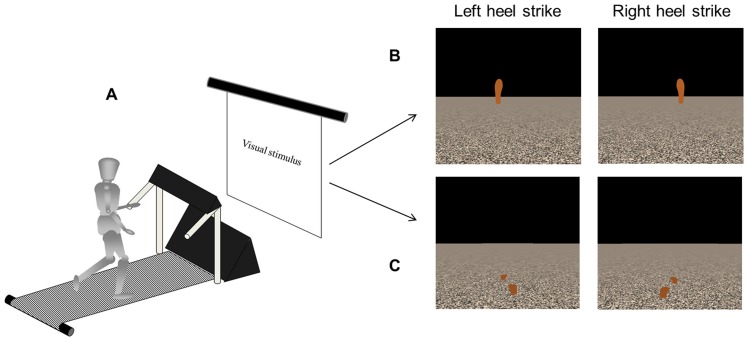
Schematic of the experimental setup in Experiment 2. While treadmill walking at a self-selected speed, the participants synchronized their heel-strike of each limb with the appearance of a corresponding virtual footprint in the virtual environment that was projected on a screen (A) that consisted of either a discrete (B) or continuous virtual stimulus (C). Both virtual environments contained a moving ground plane, providing optic flow of the environment that closely mimicked the treadmill speed.

A different visual stimulus was presented in each day and the order was counterbalanced between participants. On one test day, a discrete visual stimulus was presented, and on the other, a continuous visual stimulus was presented. Both stimuli were presented in a virtual environment on a screen in front of the treadmill, and consisted of a black sky, horizon line, and textured ground plane with identical optic flow rates (i.e., the rate of the ground plane moving toward the participants) of 1 m/s (Figure 4). The optic flow rate was set at a constant rate of 1 m/s between participants, even though the participants were allowed to self-select their walking speed. The 1 m/s optic flow rate was selected because it was near the average self-selected walking speed from Experiment 1. The discrete stimulus included two virtual footprints that alternately flashed for 200 ms at eye-height in the virtual environment (Figure 4B), whereas the continuous stimulus included two virtual footprints that continuously slid along the ground plane in an alternating fashion (Figure 4C). In the continuous stimulus, each virtual footprint started by appearing approximately 2 m in the foreground and then slid back toward the participant. Once the virtual footprint reached the edge of the screen, it reappeared in its original position and continued the sliding cycle. The virtual footprint did not include a flight phase. Thus, the sliding footprints provided near continuous information about the timing leading up to the event (appearance of the virtual footprint which prompted heel contact of the corresponding limb) by being visible throughout the majority of the gait cycle, while the discrete stimulus did not. In both stimulus types, the time between appearances of the right virtual footprint was prescribed by a fractal time series and the left virtual footprint appeared halfway through the prescribed time interval. Participants were instructed to be at right heel strike when the right virtual footprint appeared in the foreground and vice versa. The same fractal time series was used to control both stimuli, which exhibited persistence (DFA α = 0.98) and contained 500 data points that were bounded within 1.00–1.35 sec (mean 1.17±0.07 sec). The mean time in the stimuli time series in Experiment 2 was increased to more closely match the baseline stride-to-stride interval time observed in our participants from Experiment 1. However, the same structure and magnitude of variability in the stimuli time series was used for both experiments.

Identical to Experiment 1, 12 reflective markers were affixed on the lower limbs and 3D motion capture data was collected at 200 Hz (Qualisys, Gothenburg, Sweden). Markers were placed bilaterally at the mid-thigh, knee, mid-shank, ankle, heel, and toe. The sagittal knee angle was calculated from the mid-thigh, knee, and mid-shank reflectors and the stride-to-stride intervals were calculated by determining the time between peak knee flexions in each stride using a custom algorithm created in Matlab (Mathworks, Inc., Natick, MA). The stride-to-stride interval time series were separated into three phases within each stimulus type: (1) pre-synchronization, (2) synchronization, and (3) post-synchronization. Each phase of the stride-to-stride interval time series was submitted to DFA to index the presence and strength of the fractal patterns.

#### Statistics

All statistics calculated with the IBM SPSS Statistics Package (version 18, IBM Corporation, New York). Summary statistics (mean and standard deviation) and the fractal structure (DFA α) of the stride-to-stride intervals were examined. As in Experiment 1, only data from the right limb were analyzed because no difference between limbs was observed in our previous research [27] . Tests of normality (skewness, kurtosis, and Kolmogorov-Smirnov) indicated all dependent variables were normally distributed. Separate 2 × 3 (stimulus type × phase) repeated measures ANOVA were used to examine each dependent variable (*p*≤.05). Follow-up Bonferroni corrected *t*-tests were used when appropriate.

### Results

#### Summary statistics

**Figure 5:**
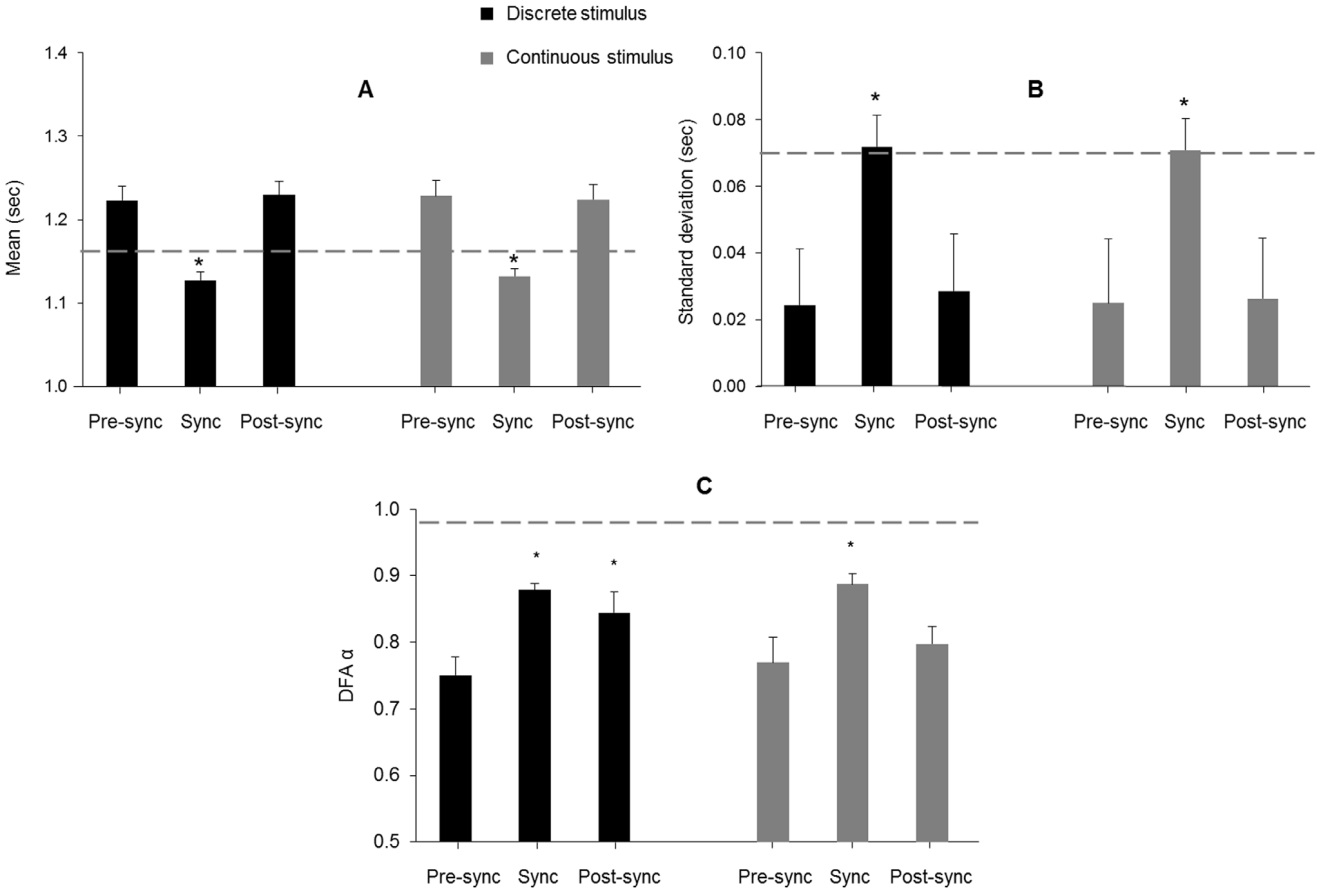
Mean, standard deviation, and DFA α of the stride interval time series in Experiment 2. A significant decrease in mean (A) and increase in standard deviation (B) was observed during the synchronization phase with both the discrete and continuous stimuli. The dashed gray line indicates the mean (1.17 sec) and standard deviation (0.07 sec) of the fractal stimulus that was used during the synchronization phase. Error bars represent standard error. Asterisks indicate the sync phase was significantly different relative to the pre- and post-sync phases. A significant increase in DFA α (C) was observed in the synchronization phase for both stimuli. However, only the discrete stimulus (black bars) led to the retention of the trained fractal structure, while the continuous stimulus (gray bars) did not lead to retention. The dashed gray line indicates the DFA α value (0.98) of the fractal stimulus that was used during the synchronization phase. Asterisks indicate the sync and post-sync phases were different than the pre-sync phase with the discrete stimulus, but only the sync phase was elevated with the continuous stimulus.

A main effect of phase was observed for the mean, *F*(2,28) = 25.9, *p*<.001, partial η^2^ = .65, and standard deviation, *F*(2,28) = 88.1, *p*<.001, partial η^2^ = .86), of the stride-to-stride intervals. Follow-up tests indicated that the mean and standard deviation in the pre-synchronization and post-synchronization phases were not different, but the synchronization phase had a significantly lower mean and higher standard deviation in both stimulus types (*p*<.001; Figure 5). There were no significant differences for stimulus type mean (*p* = .699) and standard deviation (*p* = .466), or for the phase × stimulus type interaction for mean (*p* = .491) and standard deviation (*p* = .451).

#### Fractal structure

**Figure 6:**
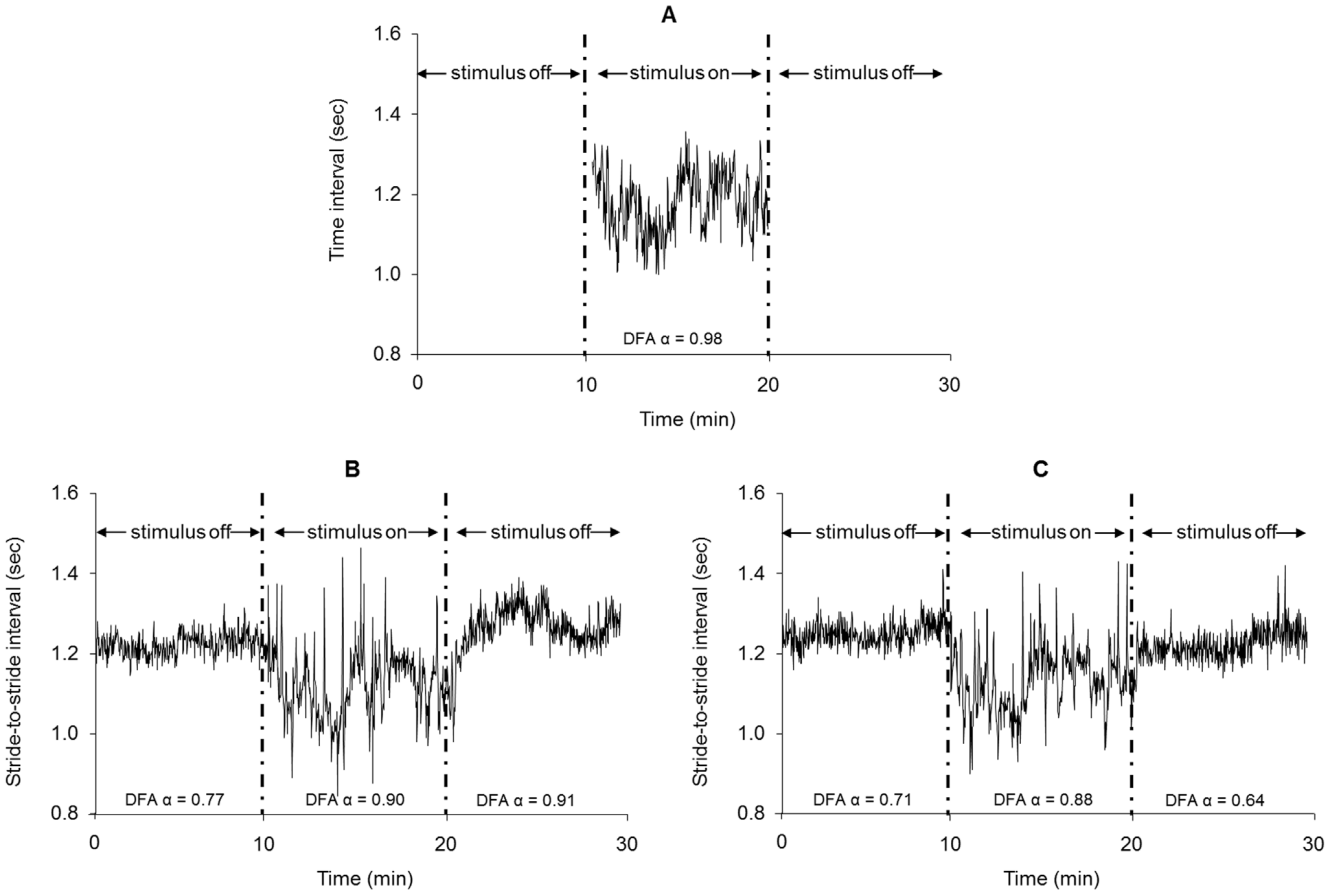
Time series of the stimulus and stride intervals in Experiment 2. The fractal time series used to drive both stimuli (A) and one participant's stride interval time series before, during, and after synchronizing with the discrete stimulus (B) and the continuous stimulus (C). DFA α increased in the synchronization phase with both stimuli, but only remained elevated in the post-synchronization phase when the discrete stimulus was employed.

The fractal structure of the time series prescribing the appearance of the right virtual footprint in both stimuli, along with the stride-to-stride intervals of the right limb for one participant in the pre-synchronization, synchronization, and post-synchronization phases in each stimulus type are shown in Figure 6. A significant main effect of phase was observed for DFA α of the stride-to-stride intervals, *F*(2,28) = 16.8, *p*<.001, partial η^2^ = .55. Follow-up tests indicated that DFA α increased in the synchronization phase in both stimulus types (*p*<.002). Importantly, in the post-synchronization phase DFA α remained elevated in the discrete stimulus (*p* = .009) compared to the pre-synchronization phase, but returned to the pre-synchronization level in the continuous stimulus (*p* = .228; Figure 5). The stimulus type main effect (*p* = .406) and phase × stimulus type interaction (*p* = .296) were not significant.

## Discussion

These experiments replicated previous findings showing that fractal gait patterns shift in a predictable direction when participants synchronize their gait cycle to a fractal stimulus [20], [27], [28], [29], [30]. The purpose of the current experiment was two-fold: (1) to determine if the new fractal gait patterns are retained after the fractal stimulus is removed and (2) to determine if the manner in which the fractal intervals were presented (discrete or continuous stimulus) affect the strength and retention of the fractal gait patterns. Experiment 1 showed that fractal gait patterns are retained up to 15 minutes after the stimulus was removed, supporting our first hypothesis. Our second experiment showed that both a continuous and discrete fractal stimulus could lead to more persistent gait patterns, but the adopted fractal patterns were only retained when the discrete stimulus was used. These results only partially supported our second hypothesis.

Visual or auditory stimulus synchronization is a relatively common method to study the neuromotor properties of timing [34], [35], [36], [37], [38], [39]. However, in all of those studies the stimulus that primed the timing behavior (typically finger tapping or circle drawing) exhibited a constant interval between beats. Given that fractal behavior emerges once the stimulus is removed [40], [41], [42], a stimulus that incorporates fractal patterns may be more useful in the examination of the neuromotor properties of timing. To that end, researchers have begun employing fractal stimuli to discover how timing emerges in a variety of tasks and to examine the flexibility of timing control [20], [27], [28], [30], [43], [44]. A concept supporting much of this research is that of strong anticipation, which suggests that fractal behavior emerges from the individual's perception of the fractal properties of the stimulus [45], [46], [47]. Thus, the desired fractal behavior of the participant can be manipulated in specific ways, so long as the task requirements are attainable. For example, participants were able to produce a variety of fractal patterns in their finger tap intervals when the fractal properties of a visual stimulus were manipulated [44]. This finding was extended to the timing in stride-to-stride intervals in our previous work, and indicated that a fractal visual stimulus could be used to shift fractal gait patterns toward persistence or randomness [27]. Since natural aging and pathology can shift fractal gait patterns toward randomness, we focused on developing and retaining persistence in Experiment 1 and provided evidence that persistent fractal gait patterns are retained for up to 15 minutes after stimulus removal. Further, we showed that the fractal patterns are not being driven by the immediate locomotor behavior following stimulus removal (minutes 1–5). This expands the work by Hove et al. [20] and Uchitomi et al. [30], which showed that Parkinson's patients who adopted fractal gait patterns from a fractal auditory stimulus retain the patterns for three minutes. The findings of Experiment 1 in the current study and from earlier work [20], [30] suggest that the production of fractal gait patterns is not merely a consequence of synchronizing to a fractal metronome. Rather, a reorganization of the neuromotor coordination pattern may be occurring, allowing for the retention of the fractal gait patterns after the stimulus is taken away. Future work in this area should focus on the frequency, intensity, and duration needed to expand the retention effect across multiple days and weeks.

The metronome time series had a smaller mean and larger standard deviation than was exhibited in the participants' baseline gait behavior in both experiments. This required the participants to exhibit faster and more variable strides during the synchronization phase to ensure that they expended effort during task performance. The stride interval mean and standard deviation of participants closely mimicked the mean and standard deviation of the metronome, indicating that the participants were able to synchronize their gait cycle to comply with both the magnitude (mean and standard deviation) and structure (DFA α) of the variably timed metronome. These results are congruent with recent research by Marmelat et al. showing that participants are able to entrain their gait dynamics to a stimulus when the magnitude or structure of variability of a fractal auditory metronome are manipulated [29].

In both our previous work [27] and in Experiment 1 of our current study, the fractal properties of the stride-to-stride intervals fell short of the fractal properties prescribed by the stimulus; a finding incongruent with earlier research that examined fractal timing properties in finger tapping [44]. This could be due to a number of factors: (1) the increased complexity of controlling gait compared to finger tapping (i.e., increased degrees of freedom to control), (2) mechanical factors, such as increased lower limb inertia relative to the inertia of a finger, (3) spatial constraints imposed while walking on a treadmill or (4) difficulty perceiving fractal timing properties from a discrete stimulus. To examine the last potential factor and determine if fractal gait patterns could be more precisely shifted in a desired direction, we elected to investigate whether a continuous visual stimulus influenced the corresponding gait behavior in Experiment 2 of the current study.

Previous work that examined the nature of visual and auditory stimuli to facilitate synchronization suggests that a stimulus containing continuous information preceding the event leads to enhanced synchronization compared to a stimulus that only provides discrete information (i.e., the event only) [33]. In our previous study [27] and in Experiment 1 of the current study, the fractal visual stimulus only provided discrete information that indicated when the participant was to be at heel contact. This information was presented with a visual stimulus that flashed at various time intervals on a screen in front of a treadmill while the participant was walking. This method did not provide the participant with any visual information about when the flash was going to occur. Given the difficulty of synchronizing to a fractal metronome while treadmill walking, it is not surprising that participants' fractal gait patterns fell short of the prescribed behavior from the visual stimulus when presented discretely. Since visuomotor synchronization has been shown to increase with a continuous stimulus [33], we modified our visual stimulus so that near continuous visual information about the fractal timing between events was available in Experiment 2. This was done via two virtual footprints (one for each leg) that alternately slid along the ground plane until they reached the end of the screen. At that point, the footprint would reappear in the foreground and then slide backward again. Thus, participants were provided with visual information throughout most of the stride that corresponded to different phases of their gait cycle to ensure that participants were at heel contact at the specified time. No difference between stimulus type was observed in the synchronization phase in Experiment 2, and this indicated that the continuous visual stimulus did not enhance the strength of the fractal gait patterns compared to the discrete visual stimulus. It is plausible that the lack of a flight phase in the sliding footprints broke up the continuity of the continuous stimulus, thereby leading to gait behavior consistent with discrete stimulus entrainment. The fractal patterns in the synchronization phase in both stimulus types were nearly identical to our previous study [27], and also to Experiment 1 in the current study. Furthermore, the developed fractal gait patterns were retained with the discrete stimulus, replicating the results from Experiment 1. However, retention was not observed with the continuous stimulus. This is particularly interesting because the magnitude of variability (i.e., standard deviation) was not different in the post-synchronization phases in the two stimulus types, but the structure of variability was, potentially highlighting a reorganization of locomotor control.

The difference in retention of fractal behavior in the two stimulus types may have been due to the attention required to complete the task during the synchronization phase. Since the discrete stimulus offered only information about the event, it is plausible that participants may have more actively attended to the overall timing structure between the events. With the continuous stimulus, visual information was available throughout most of the stride, thereby off-loading the cognitive demand to the task and potentially requiring less attention from the participant. Since fractal gait patterns were shifted toward persistence in both stimulus types in the synchronization phase, but only retained with the discrete stimulus, it is plausible that different strategies were used in each of the stimulus types. Even though synchronizing to both visual stimuli led to altered fractal behavior, the discrete visual stimulus may have provided information that was conducive for the reorganization of locomotor control.

An equally plausible case for these divergent findings could be made based on the idea of constraints. The discrete stimulus dictated heel strike times but allowed the participant to freely vary their movements in an individualized way so that there were myriad movement patterns that led to heel synchronization. Conversely, the continuous stimulus more rigidly defined the gait timing and, thus, forced each participant into a more constrained movement pattern throughout the gait cycle. Constraining gait in this way may not allow the locomotor system to search for and converge on a preferred organization for task completion. Instead, the system may have been forced into an organization that, while adequate for task completion, was not as robust and therefore did not persist once the stimulus was removed. Future studies should examine this question in more detail given its potential implications for the rehabilitation of patients with locomotor deficits. Measures of coordination between limbs that allow for the identification of attractor states (i.e., stable solutions of gait dynamics) could be useful in this pursuit. Such measures would allow for the characterization of different organization patterns, and the stability of such patterns, in the context of a synchronization task such as this.

## Conclusions

The concept of developing specific patterns of variability in gait is gaining favor in the literature because of its potential to positively enhance gait functionality [20], [27], [28], [29], [30]. The current experiments examined whether more persistent gait patterns are retained after entrainment and also whether a continuous or discrete stimulus was more appropriate for adopting and retaining fractal gait patterns. The data indicated that fractal patterns are indeed retained up to 15 minutes after stimulus removal. Our results also demonstrated that both a discrete and continuous stimulus are viable tools to alter fractal patterns in gait during synchronization. However, retention of the fractal gait patterns was only observed following the discrete stimulus. This information, in conjunction with previous findings in this domain [26], has begun to lay the foundation for the use of fractal stimuli to alter specific fractal behavior for the restoration of adaptive, functional movement patterns during locomotion.
